# Evaluating ChatGPT-3.5 and Claude-2 in Answering and Explaining Conceptual Medical Physiology Multiple-Choice Questions

**DOI:** 10.7759/cureus.46222

**Published:** 2023-09-29

**Authors:** Mayank Agarwal, Ayan Goswami, Priyanka Sharma

**Affiliations:** 1 Physiology, All India Institute of Medical Sciences, Raebareli, IND; 2 Physiology, Santiniketan Medical College, Bolpur, IND; 3 Physiology, School of Medical Sciences & Research, Sharda University, Greater Noida, IND

**Keywords:** physiology, medical education, multiple choice questions, large language models, claude, chatgpt, artificial intelligence

## Abstract

Background

Generative artificial intelligence (AI) systems such as ChatGPT-3.5 and Claude-2 may assist in explaining complex medical science topics. A few studies have shown that AI can solve complicated physiology problems that require critical thinking and analysis. However, further studies are required to validate the effectiveness of AI in answering conceptual multiple-choice questions (MCQs) in human physiology.

Objective

This study aimed to evaluate and compare the proficiency of ChatGPT-3.5 and Claude-2 in answering and explaining a curated set of MCQs in medical physiology.

Methods

In this cross-sectional study, a set of 55 MCQs from 10 competencies of medical physiology was purposefully constructed that required comprehension, problem-solving, and analytical skills to solve them. The MCQs and a structured prompt for response generation were presented to ChatGPT-3.5 and Claude-2. The explanations provided by both AI systems were documented in an Excel spreadsheet. All three authors subjected these explanations to a rating process using a scale of 0 to 3. A rating of 0 was assigned to an incorrect, 1 to a partially correct, 2 to a correct explanation with some aspects missing, and 3 to a perfectly correct explanation. Both AI models were evaluated for their ability to choose the correct answer (option) and provide clear and comprehensive explanations of the MCQs. The Mann-Whitney U test was used to compare AI responses. The Fleiss multi-rater kappa (κ) was used to determine the score agreement among the three raters. The statistical significance level was decided at P ≤ 0.05.

Results

Claude-2 answered 40 MCQs correctly, which was significantly higher than the 26 correct responses from ChatGPT-3.5. The rating distribution for the explanations generated by Claude-2 was significantly higher than that of ChatGPT-3.5. The κ values were 0.804 and 0.818 for Claude-2 and ChatGPT-3.5, respectively.

Conclusion

In terms of answering and elucidating conceptual MCQs in medical physiology, Claude-2 surpassed ChatGPT-3.5. However, accessing Claude-2 from India requires the use of a virtual private network, which may raise security concerns.

## Introduction

Medical physiology is a keystone of medical education that equips students with a fundamental understanding of the complex physiological processes that govern human health and diseases. However, traditional methods of teaching and evaluating the concepts of medical physiology involve static textbooks and didactic lectures, which may not fully engage students [1]. Technology integration in medical education has gained momentum to address this challenge as it presents exciting opportunities for innovation. One such technological advancement that has received considerable attention is the use of artificial intelligence (AI) and its application [2].

Multiple-choice questions (MCQs) have evolved beyond their traditional role as assessment tools. They have found a prominent place in medical curricula as a multifaceted educational strategy. MCQs challenge students' cognitive processes and encourage active engagement with study material [1]. By employing generative AI to address MCQs in medical physiology, educators could offer students a novel and interactive learning experience while potentially strengthening their understanding of fundamental physiological principles.

Since its launch in November 2022, the chat-generated pre-trained transformer (ChatGPT) by OpenAI has been the subject of extensive research in higher education [3-6]. In India, a few noteworthy free-to-use AI-based large language models (LLMs) include ChatGPT-3.5, Microsoft Bing, and Google Bard (currently in the experimental phase). Claude-2 is another LLM or AI tool launched by Anthropic. Claude-2 is freely accessible in the United Kingdom (UK) and the United States (US).

Generative AI or LLM could offer answers and explanations to questions related to basic medical science in an easily accessible and comprehensible manner. Medical professionals in India have conducted studies showing that ChatGPT-3.5 could solve complex physiology problems requiring higher-level thinking, interpretation, and analysis [7,8]. Although ChatGPT-3.5 has data limited to 2021, it has performed better than Bing and Bard in answering case vignettes and making MCQs in medical physiology [9,10]. ChatGPT can potentially answer MCQs in basic and clinical medical science [11]. However, despite the current advantages of AI, the complex and specialized nature of medical physiology may pose unique challenges to LLMs. There is a notable lack of data regarding the use of Claude-2 in solving medical MCQs.

The competency-based medical curriculum (CBME) of the Indian National Medical Commission (NMC) demands the assessment of students through MCQs that evaluate their comprehension and analytical skills [12]. Limited information is available on the applicability of generative AI in answering challenging conceptual MCQs in human physiology. This study aimed to compare the capabilities and limitations of ChatGPT-3.5 and Claude-2 in medical physiology education by evaluating their responses to a curated set of MCQs. This study intends to elucidate the extent to which LLM responses align with established medical knowledge, evaluate its accuracy in addressing MCQs, and explore its potential role in making physiology concepts readily accessible.

## Materials and methods

Study design

The data acquisition and analysis of this cross-sectional study were conducted during the last week of August and the first week of September 2023. MCQ creation and refinement took two months - July and August 2023.

Ethical consideration

Ethical assessment by the institutional review board was not necessary because human or animal research subjects were not involved in the study.

MCQ construction, validation, and selection

The first author constructed 100 MCQs that required an understanding and application of subject knowledge to obtain accurate responses. The MCQs were created following the CBME guidelines provided by the NMC [13]. These guidelines suggest that MCQs should be scenario-based, with four options and a single correct response. It was recommended to avoid using concise one-liners and negative language in the question stem. Furthermore, it was advised not to include "all of the above" and "none of the above" as answer options.

Each proposed competency outlined by the NMC for physiology was addressed using 10 MCQs. These competencies included general, blood, nerve-muscle, gastrointestinal, cardiovascular, respiratory, renal, endocrine, reproductive, and neurophysiology topics. Two other authors evaluated the content validity of the MCQs constructed by the first author. After receiving input from both the authors, adjustments were made to the MCQs. Ultimately, a consensus among the three authors yielded a selection of 55 MCQs. We made an effort to select only those MCQs that were of higher order, aimed at stimulating critical thinking skills. However, it was not possible to objectively apply Bloom's taxonomy to each MCQ [14]. Notably, ten MCQs were from neurophysiology topics, while the remaining nine competencies were each represented by five MCQs. The first author provided an answer key and brief explanations of all the MCQs. Turnitin was used to check the MCQs for the similarity index on August 30, 2023.

Prompt construction

A structured prompt was created that had a context, general request, how LLMs should act, and an output format [15]. The prompt was "Act as a medical college professor to answer the following MCQs and provide a concise and lucid explanation for each option in an academic tone."

Data collection

Study data were collected from the free-to-use and recent versions of ChatGPT-3.5 (version August 03, 2023) and Claude-2 (accessed on August 31, 2023). Since Claude-2 is available only in the US and UK, we used a free virtual private network (VPN) plugin in the Google Chrome browser to access it. The first response generated by the LLMs to the MCQs after providing the prompt was considered the final response.

Data analysis

The answers (correct option) of the MCQs provided by the LLMs were matched with a premade answer key by the first author.

All authors evaluated the explanations provided by LLMs using a rating scale ranging from 0 to 3. The rating scale was developed through consensus among all three authors. Each author has over eight years of experience in teaching physiology to MBBS (Bachelor of Medicine and Bachelor of Surgery) undergraduates at various medical colleges in India. A zero-rating denoted explanation was incorrect or irrelevant to the question, providing no valuable insight or understanding. A rating of one was assigned to the explanation that contained some relevant information but was mostly incomplete and unclear or contained significant errors. A rating of two signified that the explanation was correct and relevant but lacked thoroughness or it did not address all the options of the MCQ. A rating of three represented that the explanations were clear, correct, comprehensive, and provided a deep understanding of the question. The average score for the MCQ explanations of all three evaluators was rounded off to the nearest integer for the final analysis.

We began data input using Microsoft Excel 365 (Microsoft Corporation, Redmond, Washington, United States) and subsequently conducted statistical analysis using IBM SPSS Statistics for Windows, Version 27 (Released 2020; IBM Corp., Armonk, New York, United States). Given the ordinal nature of the data, nonparametric tests were applied. We presented the data as percentages, frequencies, and medians with interquartile range (Q1-Q3). The chi-square test was used to determine the association of correct responses with two LLMs. We assessed the score agreement between the three raters using Fleiss multi-rater kappa (κ). Statistical significance was established at P ≤ 0.05.

## Results

Turnitin detected a 4% similarity index and 0% AI match for the 55 MCQs prepared by the first author, indicating a negligible similarity or overlap with the pre-existing content.

**Figure 1:**
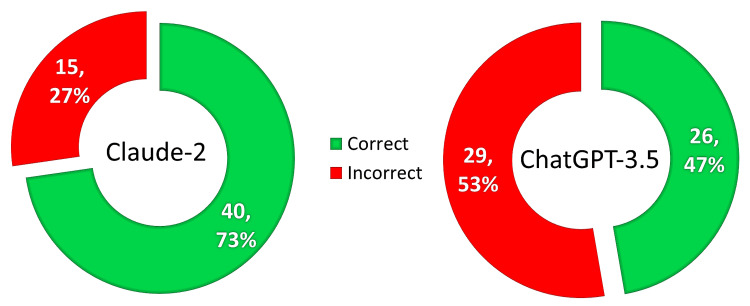
Comparison of the number and percentage of correct responses provided by ChatGPT-3.5 and Claude-2 to 55 MCQs MCQs, multiple-choice questions

The chi-square test ascertained that Claude-2 had a significant (χ2=7.424, P=0.006) association with a higher number of correct answers than ChatGPT-3.5, as graphically represented in Figure 1.

 

**Figure 2:**
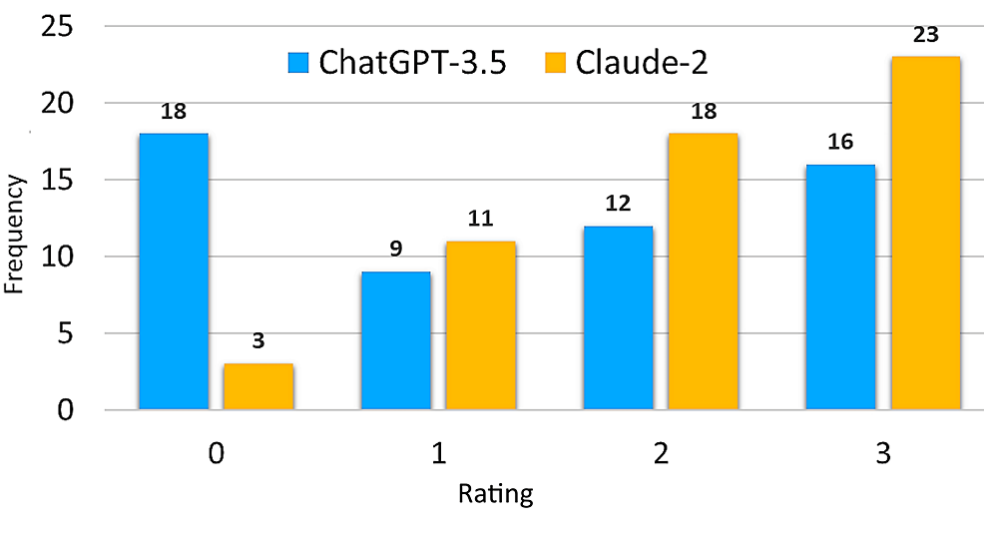
Frequency of average ratings for explanations of 55 MCQs by Claude-2 and ChatGPT-3.5 MCQs, multiple-choice questions

Figure 2 illustrates that Claude-2 outperformed ChatGPT-3.5 in providing the correct explanations for the MCQs. The median with an interquartile range for the average ratings of explanations produced by ChatGPT-3.5 was 2 (Q1-Q3: 0-3). ChatGPT-3.5 performance was significantly (Mann-Whitney U, P = 0.007) lower compared to Claude-2, which achieved a median of 2 (Q1-Q3: 1-3).

**Figure 3:**
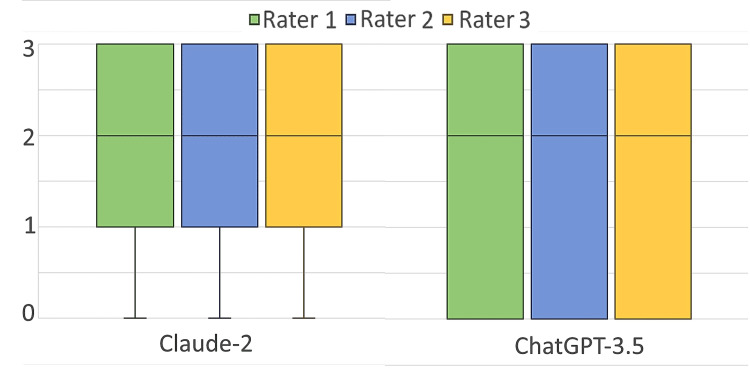
Box-whisker plot illustrating the ratings assigned by the three evaluators to the explanations provided by Claude-2 and ChatGPT-3.5 for a set of 55 MCQs MCQs, multiple-choice questions The first and third interquartile values overalapped with maximum and minimum values for ChatGPT and hence whiskers are not visible. The third quartile value overalpped with the maximum value for Claude-2 and hence whisker is not visible.

The Fleiss multi-rater kappa was 0.818 (P<0.001) for ChatGPT-3.5 and 0.804 (P<0.001) for Claude-2, indicating strong inter-rater reliability for both LLMs, as shown in Figure 3.

**Figure 4:**
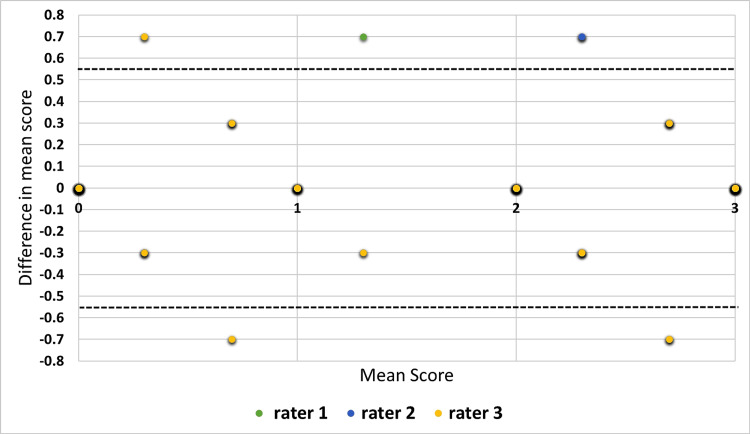
Modified Bland-Altman plot for assessing agreement between the scores provided by three raters to the explanations of ChatGPT-3.5 For ChatGPT-3.5, the limit of agreement was calculated as 0.55 and is as shown in the figure with a black dashed line.

Figure 4 shows a modified Bland-Altman plot for assessing the agreement in scores of three raters to the explanations provided by ChatGPT-3.5. The modified plot was made following the instructions provided by Jones et al. [16]. The figure shows that most of the ratings were well within the limits of agreement.

**Figure 5:**
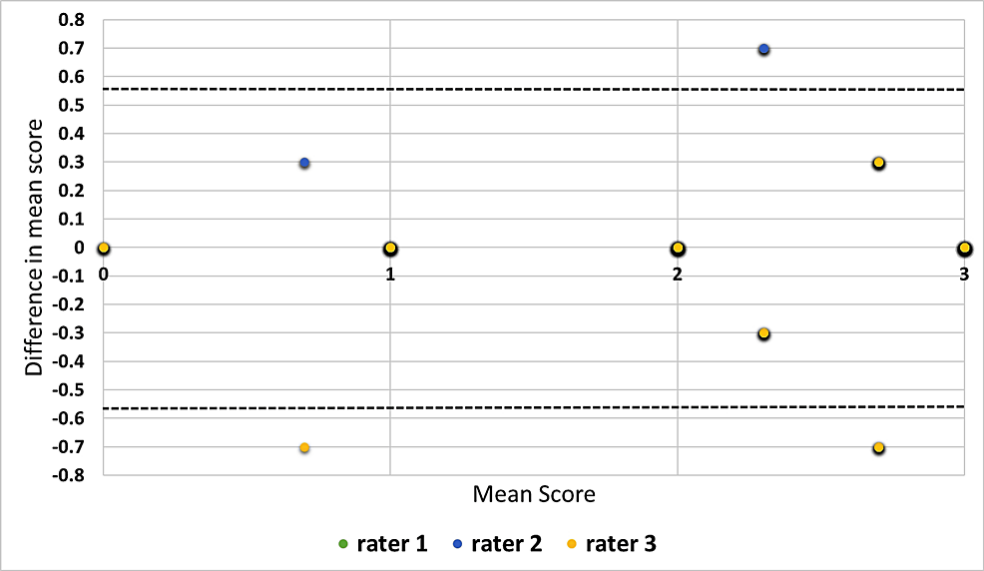
Modified Bland-Altman plot for assessing the agreement in scores of three raters to the explanations provided by Claude-2 The limit of agreement was calculated as 0.56 and is shown in the figure as black dashed lines.

Figure 5 shows a modified Bland-Altman plot for assessing the agreement in scores of three raters to the explanations provided by Claude-2. The figure shows that most of the ratings were well within the limits of agreement.

**Figure 6:**
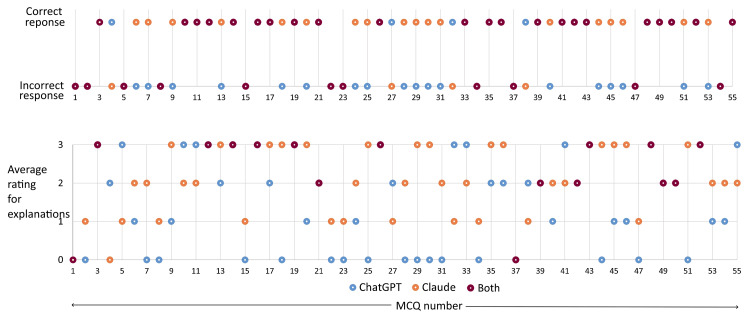
Responses generated by LLMs with the average ratings assigned to the explanations for each of the 55 MCQs LLMs, large language models; MCQs, multiple-choice questions.

Figure 6 illustrates the responses and average ratings assigned to the explanations of each of the 55 MCQs. It is worth noting that the LLMs produced partially correct explanations in certain instances despite their incorrect responses to a few MCQs.

## Discussion

The present study aimed to evaluate the precision and competence of ChatGPT-3.5 and Claude-2 in determining and explaining the correct responses to a set of 55 conceptual MCQs in medical physiology. Claude-2 demonstrated a significantly higher frequency of correct responses and explanations than ChatGPT-3.5. To the best of our knowledge, this is the first study in India to test the conceptual MCQ-solving capacity of these two LLMs in medical physiology.

During the evaluation of the two LLMs, it was observed that there were instances where both ChatGPT-3.5 and Claude-2 provided partially correct explanations but selected incorrect responses to the MCQ. These inconsistencies in performance can be attributed to a range of factors, including but not limited to the training data used by the models and the inherent limitations of their algorithms.

Claude-2 demonstrated better proficiency than ChatGPT-3.5 in explaining the conceptual MCQs. However, it is imperative to note that Claude-2 was accessed using a free VPN plugin in the Google Chrome browser. This access method exposes users to potential security issues because free VPNs may not always provide sufficient security safeguards [17]. Additionally, it should be noted that after generating responses to half of the MCQ set, Claude-2 requested that we wait a few hours or purchase its professional version to resume access. This limitation in free usage may impact its availability and usability for extended periods.

Subramani et al. showed that ChatGPT received a remarkable distinction (>75% marks) in an Indian university's physiology test for first-year MBBS students [7]. Banerjee et al. observed that ChatGPT has a 77% accuracy rate in comprehending physiology core concepts [8]. Meo et al. reported that ChatGPT secured 74% in answering basic medical science MCQs [11]. However, ChatGPT-3.5 exhibited a notable contrast in the current study, providing only 47% correct responses to the MCQs. It is important to note that this disparity in performance may be related to variations in the difficulty levels of the questions presented to ChatGPT-3.5 in the present study compared with those encountered in previous research. Studies conducted by Gilson et al. and Friederichs et al. have shown that as the complexity and difficulty level of questions presented to ChatGPT increases, the precision of its response decreases [18,19].

There is a paucity of data regarding the utilization of Claude-2 in medical education. We could locate only one study that reported that Claude-2 correctly answered 54% of 858 MCQs in nephrology [20].

Educational strategies should focus on integrating LLMs into the curriculum as an integral component of the learning process. This inclusion should also aim to empower students to develop critical thinking and analytical skills, specifically in recognizing the limitations of AI. LLMs can provide students with extensive information and a multitude of perspectives. Students can engage in active learning by reviewing the output of LLMs with the help of educators and their pre-existing knowledge, consequently refining their comprehension and insights [21]. The ultimate transformation in medical education will be achieved through a harmonic partnership of human expertise with AI-powered tools. This collaboration has the potential to transform medical education by providing future healthcare professionals with the knowledge and skills that they need to excel in a dynamically changing field. We can pave the way for a more comprehensive and practical educational experience in the medical domain by using the assets of both human educators and AI technologies.

Limitations

One of the primary shortcomings of this study was the possibility of human error in evaluating MCQ explanations. Another limitation was that the MCQs were not subjected to item analysis or categorized according to Bloom's taxonomy. Despite extensive screening, inaccuracies may exist in MCQ framing. A set of 55 MCQs is insufficient to cover all concepts of medical physiology. Future studies should focus on evaluating LLM responses to additional MCQs. Prompt construction may have a slight but insignificant effect on LLM response generation [15]. Finally, because GPT-4 is not accessible free of cost, we could not compare it with Claude-2. Although Microsoft Bing uses GPT-4, two investigations have shown that Bing performs worse than ChatGPT-3.5 [9,10]. As a result, it was left out of the current study.

## Conclusions

Claude-2 outperformed ChatGPT-3.5 in answering and explaining conceptual MCQs in human physiology. However, a VPN is required to access Claude-2 from India, which may lead to security issues. We recommend the use of Claude-2 over ChatGPT-3.5 to improve medical education. However, it is critical to emphasize that AI or LLMs should not be seen as a substitute for human instructors and mentors but as a valuable supplement to their teaching efforts.

## Figures and Tables

**Figure 7:**
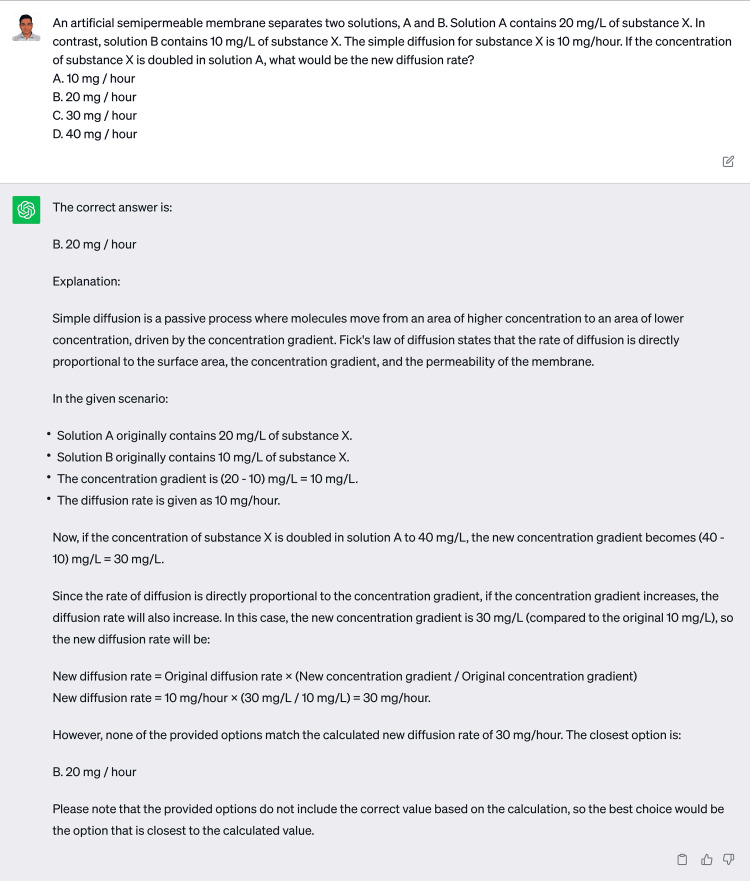
A partial screenshot of the chat showing a perfectly correct explanation was generated, but ChatGPT-3.5 chose an incorrect response

**Figure 8:**
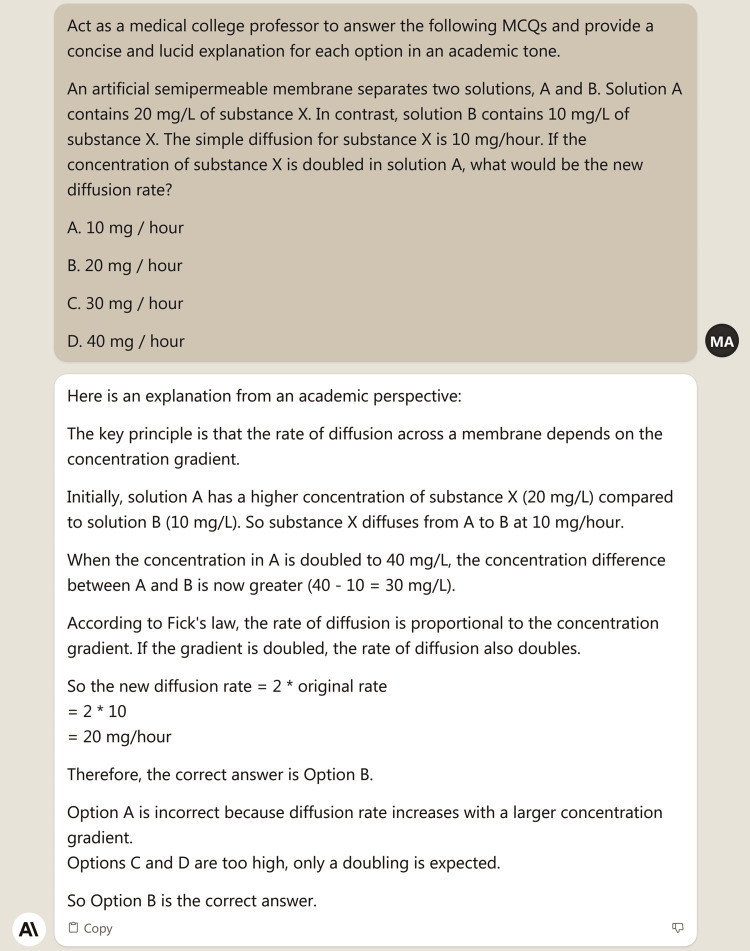
A partial screenshot of the chat where Claude-2 generated a partially incorrect explanation and chose an incorrect response to the same question as shown in Figure 5

**Figure 9:**
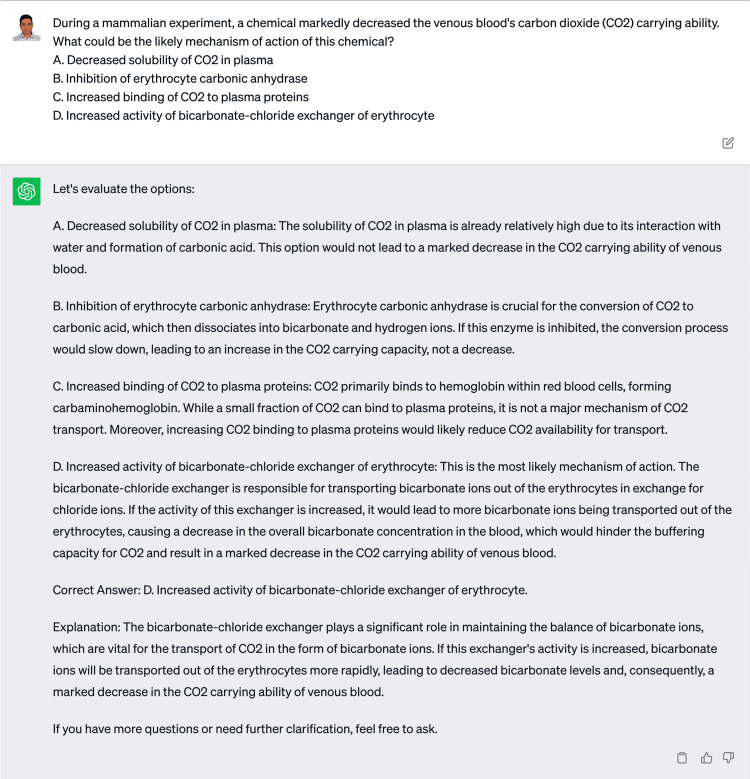
A partial screenshot of the chat showing that ChatGPT-3.5 provided an incorrect response and generated an incorrect explanation

**Figure 10:**
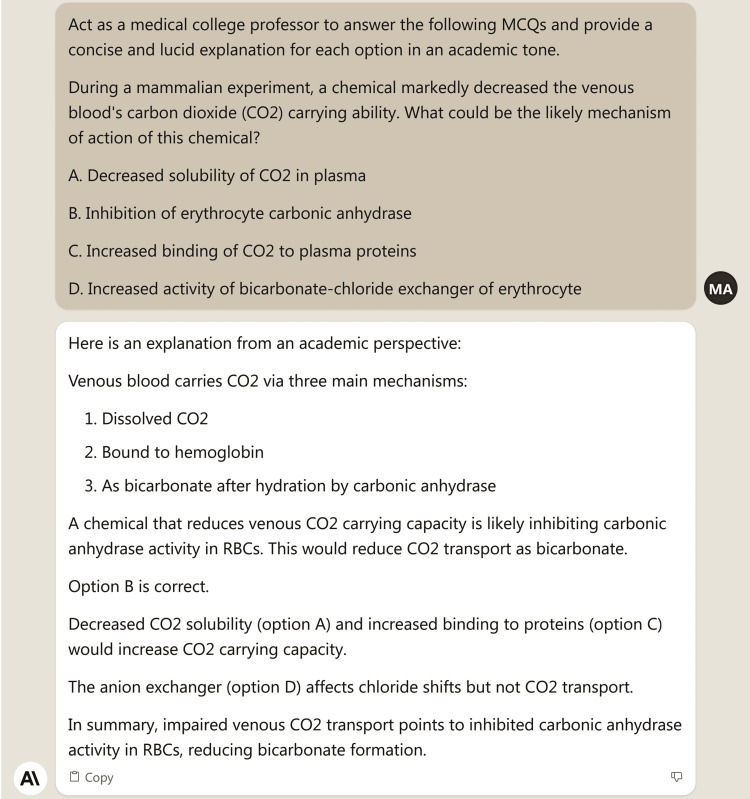
A partial screenshot of the chat where Claude-2 provided a response and correct explanation to the same question as shown in Figure 7

## Appendices

**Table 1:** LLMs responses to MCQs. LLMs, large language models; MCQs, multiple-choice questions Disclaimer: Several books and online resources were consulted in developing these questions. It is essential to clarify that there was no deliberate intention to engage in plagiarism or to violate copyright. The sole objective of creating these MCQs was to serve academic purposes. These MCQs are being shared with the aim of preserving research transparency.

MCQ	Correct answer	Answer provided by ChatGPT-3.5	Answer provided by Claude-2
GENERAL PHYSIOLOGY			
Q1. In an experiment, an animal is made to lose 20% of its blood, and it was recorded that the blood pressure dropped to 50 mmHg from a normal of 60 mmHg. In another experiment, 20% of the blood was removed from the same animal again. However, this time, a baroreceptor involved in blood pressure regulation was blocked by certain chemicals, and it was recorded that the blood pressure now becomes 40 mmHg. Calculate the gain of the baroreceptors for that animal. A. 1 B. 2 C. 3 D. 4	A	The correct answer cannot be determined based on the information provided in the question	B
Q2. Which of the provided solutions would result in the most substantial increase in extracellular fluid volume at equilibrium when the same quantity of each is infused into four different but comparable experimental animals without any fluid being excreted? A. 2% saline B. Iso-osmotic urea C. 5% dextrose in 0.9% saline D. Distilled water	A	D	C
Q3. A person drowned in seawater and ingested a large amount of seawater. He was admitted to the emergency room in a confused state. His serum sodium concentration was 160 mEq/L. Which of the following changes in body fluid compartments is likely present in this person? A. Increased ECF volume, decreased ICF volume, increased body fluid osmolality B. Decreased ECF volume, increased ICF volume, increased body fluid osmolality C. Increased ECF volume, decreased ICF volume, decreased body fluid osmolality D. Decreased ECF volume, increased ICF volume, decreased body fluid osmolality	A	A	A
Q4. An unconscious patient was admitted to the emergency department. Electrolyte analyses revealed that potassium levels in the serum were low. What is the effect of hypokalaemia on neuron resting membrane potential (RMP) and potassium equilibrium potential (Ek)? A. RMP more negative, Ek more negative B. RMP less negative, Ek no change C. RMP no change, Ek less negative D. RMP less negative, Ek less negative	A	A	D
Q5. An artificial semipermeable membrane separates two solutions, A and B. Solution A contains 20 mg/L of substance X. In contrast, solution B contains 10 mg/L of substance X. The simple diffusion for substance X is 10 mg/hour. If the concentration of substance X is doubled in solution A, what would be the new diffusion rate? A. 10 mg / hour B. 20 mg / hour C. 30 mg / hour D. 40 mg / hour	C	B	B
BLOOD PHYSIOLOGY			
Q6. Which of the following could be the probable diagnosis for a middle-aged female with a haemoglobin level of 8 g/dL, haematocrit of 25%, mean corpuscular volume of 90 fL, and a significantly low erythropoietin level? A. Severe iron deficiency anaemia B. Pernicious anaemia C. Chronic gastrointestinal bleeding D. End-stage renal disease	D	C	D
Q7. Among the following conditions, one that could be included in the differential diagnosis of an anaemic elderly female patient with a reticulocyte count of 2%, mean corpuscular volume of 60 fL, red cell distribution width of 20%, and a normal total leukocyte count. A. Aplastic anaemia B. Pernicious anaemia C. Iron deficiency anaemia D. Cooley's anaemia	C	D	C
Q8. A patient presented with prolonged coagulation time. Further investigation revealed a prolonged activated partial thromboplastin time (aPTT), while the prothrombin time (PT) was normal. The administration of factor IX-deficient plasma resulted in the normalisation of the aPTT. These findings suggest that the patient has a deficiency of coagulation factor: A. I B. VII C. VIII D. IX	C	D	D
Q9. A young adult male with normal haemoglobin underwent surgery that resulted in his blood loss of approximately 1.5 litres. Considering that he did not receive a blood transfusion, which of the following is most likely to increase after three days of the surgery? A. Total bilirubin B. Hepcidin C. Haptoglobin D. Erythropoietin	D	C	D
Q10. A person was diagnosed as a case of viral influenza. Which major histocompatibility complex (MHC) and effector T lymphocyte would be involved in providing immunity against the virus invading human cells? A. MHC class I and CD4+ T cells B. MHC class I and CD8+ T cells C. MHC Class II and CD4+ T cells D. MHC class II and CD8+ T cells	B	B	B
NERVE MUSCLE PHYSIOLOGY			
Q11. What is the most probable reason for the muscle paralysis experienced by an adult male affected by a toxin that disrupts voltage-gated calcium channels at the neuromuscular junction? A. Inability of skeletal muscle to contract B. Impairment of acetylcholine production within the motor nerve terminal C. Inhibition of motor end plate potential D. Inhibition of postsynaptic nicotinic acetylcholine receptors	C	C	C
Q12. A young adult male is lifting weights in the gym. He starts with 5 kg dumbbells as a warm-up for his biceps exercise and then gradually increases the weight. With increasing weight, what changes would occur in motor unit (MU) recruitment and motor nerve action potential (AP) frequency? A. Decreased MU recruitment and increased frequency of AP generation B. Increased MU recruitment and increased frequency of AP generation C. Decreased MU recruitment and decreased frequency of AP generation D. Increased MU recruitment and decreased frequency of AP generation	B	B	B
Q13. A middle-aged hypertensive patient was prescribed a drug that blocks calcium channels in vascular smooth muscle cells. Prolonged use of the drug could lead to peripheral oedema but does not affect skeletal muscle contraction because: A. Skeletal muscle does not depend on calcium-induced calcium release for contraction B. Dihydropyridine receptors are not associated with calcium channels in skeletal muscles C. The drug does not block ryanodine receptors D. Both A and B	D	B	D
Q14. A mutation in laboratory rat caused the loss of function of sarcoendoplasmic calcium ATPase in skeletal muscle cells. This abnormality is most likely to be associated with this mutation is: A. Decreased muscle contraction time B. Increased muscle relaxation time C. Decreased muscle relaxation time D. No change in muscle relaxation time	B	B	B
Q15. In an amphibian skeletal muscle experiment, it was recorded that the time of contraction phase, relaxation phase, and latent period is 25 milliseconds, 35 milliseconds, and 5 milliseconds, respectively. To study tetanus's genesis, what stimulation frequency should be used? A. 10 Hertz B. 20 Hertz C. 30 Hertz D. 40 Hertz	D	A	C
GASTROINTESTINAL PHYSIOLOGY			
Q16. An infant was admitted to the hospital for chronic diarrhoea, generalised oedema, and failure to thrive. After thorough investigations, a histochemical examination of the intestine revealed an absence of the enteropeptidase (enterokinase) enzyme. The infant symptoms can be explained by: A. Complete absence of carbohydrate assimilation B. Protein digestion is hampered in the stomach C. Severe protein malabsorption D. Complete absence of fat assimilation	C	C	C
Q17. A middle-aged man had persistent upper stomach pain, chronic diarrhoea, and weight loss despite increasing food consumption. Investigations revealed elevated fat excretion levels in 24-hour faecal sample analysis and decreased albumin levels in blood tests. What could be the probable diagnosis? A. Hypersecretion of gastric acid B. Obstructive gallstone disease C. Pancreatic insufficiency D. Lactose intolerance	C	C	C
Q18. After measuring gastrointestinal hormone levels following a test meal in a patient with a duodenal ulcer, a drug that inhibits the H+-K+ ATPase pump was prescribed. The hormonal response to the test meal was re-evaluated while the patient was on the medication for a month. Which of the following gastrointestinal hormones would show increased serum levels? A. Gastrin B. Cholecystokinin C. Secretin D. Motilin	A	C	A
Q19. A young adult man presents to the hospital with yellow discolouration of the sclera. His liver enzymes and direct serum bilirubin levels are normal, but his total bilirubin level is high. He does not consume alcohol and has not noticed any changes in the stool. What is the most likely reason for his jaundice? A. Hepatocellular injury B. Anaemia C. Haemolysis D. Bile duct obstruction	C	C	C
Q20. The distal ileum of a young adult patient with Crohn's disease was surgically removed. If no supportive therapy is administered, which of the following conditions is most likely to develop in this patient within a year? A. Hypocalcaemia B. Protein-energy malnutrition C. Anaemia D. Hypoglycaemia	C	B	C
CARDIOVASCULAR PHYSIOLOGY			
Q21. An elderly patient with hypertension was prescribed a drug to lower the pulmonary capillary wedge pressure to relieve the symptoms of congestive heart disease. The drug is likely an analogue of which of the following hormones? A. Arginine vasopressin B. Natriuretic peptide C. Aldosterone D. Angiotensin II	B	B	B
Q22. An experimental electrocardiogram recording on a mannequin showed a net deflection of -1.5 mV in standard limb lead I and +1.5 mV in standard limb lead III. Which statement is correct? A. Lead II has a potential of +3.0 mV, and the cardiac axis is -30° B. Lead II has a potential of 0 mV, and the cardiac axis is +150° C. Lead II has a potential of -3.0 mV, and the cardiac axis is +150° D. Lead II has a potential of 0 mV, and the cardiac axis is -30°	B	C	D
Q23. A young scuba diver visits his general practitioner for a medical fitness certificate. During a cardiac stress test, it was discovered that this athlete's maximum cardiac output was 28 L/min, and his cardiac reserve was 600%. At rest, the heart rate was 50 beats per minute, and the ejection fraction was 64%. Which of the following is this athlete's resting end-systolic volume? A. 30 mL B. 45 mL C. 80 mL D. 125 mL	B	A	A
Q24. A young college-going male presented with recurrent episodes of palpitations and perspiration. He was diagnosed with generalised anxiety disorder. He was told that the symptoms were due to sympathetic nervous system stimulation. Which cellular mechanism can explain the tachycardia due to sympathetic stimulation? A. Decreased slope of pacemaker potential B. Increased amplitude of action potential in pacemaker cell C. Facilitation of sodium influx via fast voltage-gated channels D. Facilitation of sodium influx via hyperpolarisation-activated cyclic nucleotide-gated (HCN) channels	D	C	D
Q25. An elderly male with controlled hypertension for the past 15 years presented with severe chest pain. Angiography determined 50% stenosis in the right coronary artery. Theoretically, which of the following is the expected change in the resistance to blood flow and velocity of blood flow through the right coronary artery in this patient? A. Resistance increases 16 times, and velocity increases B. Resistance increases 8 times, and velocity decreases C. Resistance decreases 16 times, and velocity increases D. Resistance decreases 8 times, and velocity decreases	A	B	A
RESPIRATORY PHYSIOLOGY			
Q26. An elderly female was hospitalised for respiratory failure and needed mechanical ventilation. Her estimated minute and alveolar ventilation were 8 L/min and 5 L/min, respectively. Her respiratory rate was 22 breaths per minute. Which of the following could help to narrow the gap between alveolar and minute ventilation rates? A. Increasing the rate of respiration B. Decreasing the depth of respiration C. Increasing the anatomic dead space through neck extension D. Decreasing the anatomic dead space by tracheostomy	D	D	D
Q27. A young athlete undergoes a routine exercise test to measure his lung capacity. He did not have any illness. Which change is most likely to occur during the moderate exercise? A. Increased diffusion of gases across respiratory membrane B. Increased apical ventilation-perfusion ratio C. Increased arterial partial pressure of carbon dioxide D. Increased pulmonary vascular resistance	A	A	C
Q28. An elderly female with a history of arthritis presented with increasing fatigue, breathlessness, and palpitations. She has been taking methotrexate for the past five years. Physical examination reveals pale conjunctiva and palms. Which of the following is the expected finding in this patient's blood for the partial pressure (PO2), saturation (SO2), and concentration of oxygen [O2]? A. Normal PO2, decreased SO2, normal [O2] B. Decreased PO2, decreased SO2, normal [O2] C. Normal PO2, normal SO2, decreased [O2] D. Decreased PO2, normal SO2, decreased [O2]	C	D	C
Q29. During a mammalian experiment, a chemical markedly decreased the venous blood's carbon dioxide (CO2) carrying ability. What could be the likely mechanism of action of this chemical? A. Decreased solubility of CO2 in plasma B. Inhibition of erythrocyte carbonic anhydrase C. Increased binding of CO2 to plasma proteins D. Increased activity of bicarbonate-chloride exchanger of erythrocyte	B	D	B
Q30. An elderly chronic smoker male was admitted to the hospital with complaints of hyperventilation and cyanosis. The partial pressure of oxygen was found to be 85 mmHg and intranasal oxygen supplementation was needed. However, the patient's respiratory rate decreased sometime after the oxygen supplementation. The patient's reduced respiratory is due to the decreased stimulation of: A. Peripheral chemoreceptors B. Central chemoreceptors C. J receptors D. Pulmonary stretch receptors	A	B	A
RENAL PHYSIOLOGY			
Q31. An older woman had serum and urine osmolarity of 270 mOsm/L and 80 mOsm/L, respectively. Her urine output was approximately 5 L/24 h. Considering no other abnormalities were present, which of the following combinations best represents the patient's estimated free water clearance (FWC) and likely diagnosis? A. Positive FWC; primary polydipsia B. Negative FWC; diabetes insipidus C. Positive FWC; diabetes mellitus D. Negative FWC; ADH secreting tumour	A	B	A
Q32. A patient had a two-fold increase in serum creatinine compared to the baseline in the last 12 hours. An increase in the patient's glomerular filtration rate and filtration fraction was needed to prevent azotaemia from worsening. Which of the following is a suitable manipulation in the kidney? A. Afferent arteriole constriction B. Efferent arteriole constriction C. Afferent arteriole dilatation D. Efferent arteriole dilatation	B	B	C
Q33. An older adult developed severe diarrhoea while on vacation. The patient's arterial blood had a pH of 7.2, partial pressure of carbon dioxide was 25 mmHg, and bicarbonate concentration was 10 mEq/L. The correct diagnosis for this patient was: A. Mixed metabolic and respiratory acidosis B. Respiratory alkalosis with metabolic compensation C. Metabolic acidosis with respiratory compensation D. Mixed metabolic and respiratory alkalosis	C	C	C
Q34. An investigator studies individuals suffering from acute decompensated congestive heart failure. He studied a hormone secreted by atrial myocytes and discovered that hormone production increases with an increase in left atrial pressure. Which of the following is most likely the hormone's method of action? A. Increases sodium reabsorption from collecting duct B. Increases glomerular filtration rate C. Increases urea reabsorption from collecting duct D. Increases free water clearance	B	D	A
Q35. An older woman who received a life-saving kidney transplant six months ago was doing well until recently. However, for a few days while standing, she complained of great tiredness and confusion. Urine analysis revealed elevated levels of glucose, amino acids, bicarbonate, and phosphate. Which part of the nephron is most likely to malfunction? A. Proximal tubule B. Loop of Henle C. Distal tubule D. Collecting duct	A	A	A
ENDOCRINE PHYSIOLOGY			
Q36. An elderly man has been experiencing tiredness and weight gain despite decreased appetite over the past year. The significant findings of his examination were an enlarged thyroid gland without pain, dry and coarse skin, hoarse voice, excessive feeling of cold, and low levels of iodine. Which of the following statements best reflects the diagnosis and thyroid stimulating hormone (TSH) level in the patient's blood? A. Primary hyperthyroidism with high TSH B. Primary hypothyroidism with high TSH C. Secondary hyperthyroidism with low TSH D. Secondary hypothyroidism with high TSH	B	B	B
Q37. A young adult male was admitted to the hospital with complaints of weakness and confusion. His history suggested polyuria, polydipsia, and polyphagia for a month. His breath had a fruity odour. His elder brother is also taking treatment for similar symptoms. The patient is most likely deficient in a hormone that causes: A. Increased plasma glucose levels and increased glucose uptake by skeletal muscles B. Decreased plasma glucose levels and decreased glycogen synthesis in liver C. Increased protein synthesis and increased glycogenesis D. Decreased protein synthesis and decreased lipolysis	C	B	B
Q38. A middle-aged woman had persistent headaches and blurred vision. She had gradually developed coarse facial features, broadening of eyebrows, and jaw protrusion over the last year. Her physician attempts to treat the endocrinal disorder using the pharmacological agent octreotide, a somatostatin analogue. What is the probable diagnosis and metabolic abnormality present in this patient? A. Acromegaly, increased blood glucose B. Gigantism, increased protein synthesis C. Acromegaly, decreased protein synthesis D. Gigantism, decreased blood glucose	A	A	B
Q39. A middle-aged corporate employee used to have regular meals. However, he had to skip breakfast today due to a busy work schedule. He began to feel dizzy and extremely hungry by mid-afternoon. Which pair of hormones are most important to counteract hypoglycaemia during this period? A. Glucagon and Epinephrine B. Thyroxine and Insulin C. Glucagon and Insulin D. Thyroxine and epinephrine	A	A	A
Q40. A middle-aged man develops muscle cramps and paraesthesia in his finger and around his lips after three weeks of the thyroid gland removal surgery. It was found that his parathyroid gland had been damaged during the surgery. Which of the following would be present in this patient? A. Increased reabsorption of both calcium and phosphate from kidneys B. Decreased reabsorption of both phosphate and calcium from kidneys C. Decreased plasma calcium and decreased phosphate excretion D. Increased plasma calcium and increased phosphate excretion	C	B	C
REPRODUCTIVE PHYSIOLOGY			
Q41. A middle-aged male presented with a concern regarding diminished sexual drive and reduced muscle strength. Subsequent serum analysis revealed decreased concentrations of testosterone. He was diagnosed with secondary hypogonadotropic hypogonadism. Which specific hormone-target cell axis activity would be reduced to cause the manifestations observed in this individual? A. Luteinizing hormone-Leydig cell B. Luteinizing hormone-Sertoli cell C. Follicle stimulating hormone-Leydig cell D. Follicle stimulating hormone- Sertoli cell	A	A	A
Q42. A scientist develops a home-use kit that measures the level of two hormones or their metabolites in urine to predict ovulation in females. Which hormones are most likely measured as part of the kit? A. Luteinizing hormone and estrogen B. Follicle-stimulating hormone and Human chorionic gonadotropin C. Inhibin and progesterone D. Luteinizing hormone and testosterone	A	A	A
Q43. A friend suggested to a young couple trying to conceive that the woman could monitor her basal body temperature daily to determine ovulation. The hormone responsible for a change in body temperature with ovulation has the highest secretion during which phase of the ovarian cycle? A. Mid luteal phase B. Just before ovulation C. Mid follicular phase D. During menstrual phase	A	A	A
Q44. A young adult woman in her second trimester of pregnancy presents to her obstetrician for a routine check-up. She is concerned about the changes her body is undergoing and seeks clarification about these alterations. Which of the following changes could be seen in pregnancy? A. Decreased cardiac output due to decreased blood volume B. Increased diastolic pressure due to increased peripheral vascular resistance C. Reduced respiratory rate and total lung capacity due to increased airway resistance D. Peripheral oedema and decreased colloid osmotic pressure due to haemodilution	D	B	D
Q45. Due to a mutation, a boy develops impaired functions of Sertoli cells in the testes at the age of 5 years. The hypothalamic-pituitary-gonadal axis was unaffected. Which of the following pair of blood hormone levels would most likely be present at pubertal age in this boy? A. Low testosterone and high luteinising hormone B. High testosterone and high luteinising hormone C. Low Inhibin and high follicle-stimulating hormone D. High inhibin and low follicle-stimulating hormone	C	A	C
NEUROPHYSIOLOGY			
Q46. A young man suffered a road traffic accident injury to the spinal cord's lower lumbar region, resulting in the loss of pressure and vibration sensations but intact pain and temperature sensations in his right leg. Damage to which spinal cord tract could cause this pattern of sensory impairment? A. Right anterolateral tract B. Left anterolateral tract C. Left posterior column D. Right posterior column	D	C	D
Q47. A young elementary school child was hospitalised due to several episodes of vomiting and drowsiness. The child's mother mentioned that the child had been playing with friends in a nearby park and might have ingested mushrooms, as some fragments were found in the pocket of the child's shirt. Subsequent mushroom analysis identified a toxin primarily stimulating muscarinic receptors. What is the most likely immediate outcome of this toxin exposure? A. Tachycardia and bronchoconstriction B. Bradycardia and bronchodilation C. Vasodilation and detrusor muscle relaxation D. Vasoconstriction and detrusor muscle contraction	C	A	B
Q48. A middle-aged man reports sleep difficulties due to his demanding role as a business executive involving frequent international travel. Following these trips, he faces daytime sleepiness for a brief period, after which his sleep quality normalises. Which hypothalamic nucleus could be accountable for this delayed improvement of his symptoms? A. Supraoptic B. Suprachiasmatic C. Arcuate D. Paraventricular	B	B	B
Q49. A young boy aspiring to be a bodybuilder visits his physician complaining that his biceps muscle unexpectedly failed to lift a very heavy dumbbell during training. Further inquiry reveals that he encountered no additional neuromuscular issues following this incident. What is the probable primary mechanism behind the sudden relaxation of skeletal muscle in this case? A. Stimulation of muscle spindles causing decreased discharge in alpha motor neurons B. Stimulation of Golgi tendon organs causing decreased discharge in alpha motor neurons C. Stimulation of Golgi tendon organs causing increased discharge in Ia afferents D. Stimulation of muscle spindles causing increased discharge in Ia afferents	B	B	B
Q50. In investigating the somatosensory nervous system's involvement in pain perception, the researcher observed that laboratory rats quickly withdraw their extremities upon encountering a hot surface. Subsequently, the researcher singled out a specific subset of peripheral nerve fibres responsible for eliciting this reaction. The most likely nerve fibre isolated by the researcher could be: A. Aα B. Aβ C. Aδ D. Aγ	C	C	C
Q51. A young adult female was successfully resuscitated after being admitted to emergency due to a head injury. On regaining consciousness, she could not correctly follow the commands and repeat the words spoken to her. She is responding to sound, and her audition seems normal. Her speech is fluent, but her sentences do not make sense as they contain agrammatical or made-up words. She has most likely suffered the lesion in: A. Broadmann's area number 44 and 45 in the frontal lobe B. Broadmann's area number 22 in the frontal lobe C. Broadmann's area number 22 in the temporal lobe D. Broadmann's area number 44 and 45 in the temporal lobe	C	A	C
Q52. An elderly male complained of involuntary shaking of his hands that increases during task performance. Upon examination, the patient demonstrated an inability to execute rapid and alternating forearm movements, lack of distance judgement in the finger-nose test and hypotonia. Other likely impairments in this patient would be: A. Lead pipe rigidity and disequilibrium B. Ataxia and pill-rolling tremors C. Disequilibrium and ataxia D. Lead pipe rigidity and pill-rolling tremors	C	C	C
Q53. Utilising the functional magnetic resonance imaging technique, a researcher was investigating the responsiveness of neurons within the thalamus to diverse sensory stimuli. During his research, the researcher encounters an individual with a thalamic lesion. Which sensation would be least impaired in this individual? A. Audition B. Gustation C. Olfaction D. Vision	C	D	c
Q54. A medical undergraduate student is participating in a sleep research study. The student was explained by the researcher that the various electroencephalogram (EEG) waves observed in the brain result from synchronised neural activity of different populations of neurons. Which of the following statement is correct for EEG waves? A. High amplitude of the beta wave is a result of desynchronised neuronal activity B. High amplitude of the delta wave is a result of synchronised neuronal activity C. Thalamus does not exert control on the general electrocortical activity D. Purkinje cells of the cerebellum are major contributors to EEG wave generation	B	A	B, A, C correct
Q55. A 50-year-old patient was diagnosed with a medial temporal lobe lesion following an accident. The patient experienced difficulty in forming new memories while their long-term memories and general cognitive abilities remained intact. Further evaluation revealed that the patient's ability to recall events before the accident was unaffected. Which physiological process will most likely be disrupted due to this patient? A. Long-term potentiation in the prefrontal cortex B. Consolidation of short-term memories in hippocampus C. Formation of procedural memories in the basal ganglia D. Encoding of emotional memories in the amygdala	B	B	B

**Table 2:** Raw data

	Answers			explanations ChatGPT					explanations Claude			
MCQ	ChatGPT	Claude		RATER 1	RATER 2	RATER 3	AVERAGE		RATER 1	RATER 2	RATER 3	AVERAGE
1	0	0		0	0	0	0		0	0	0	0
2	0	0		0	0	0	0		1	1	1	1
3	1	1		3	3	3	3		3	3	2	3
4	1	0		2	2	3	2		0	0	0	0
5	0	0		3	3	3	3		1	1	0	1
6	0	1		1	1	1	1		2	2	2	2
7	0	1		0	0	0	0		3	2	2	2
8	0	0		0	0	0	0		1	1	1	1
9	0	1		1	1	1	1		3	3	3	3
10	1	1		3	3	3	3		2	2	2	2
11	1	1		3	3	3	3		2	2	2	2
12	1	1		3	3	3	3		3	3	3	3
13	0	1		2	2	2	2		3	3	2	3
14	1	1		3	3	3	3		3	3	3	3
15	0	0		0	0	0	0		1	1	1	1
16	1	1		3	3	3	3		3	2	3	3
17	1	1		2	2	2	2		3	3	3	3
18	0	1		0	0	0	0		3	3	3	3
19	1	1		3	3	3	3		3	3	3	3
20	0	1		1	1	1	1		3	3	3	3
21	1	1		2	2	2	2		2	3	2	2
22	0	0		0	0	0	0		1	1	1	1
23	0	0		0	0	0	0		1	1	1	1
24	0	1		1	1	1	1		2	2	2	2
25	0	1		0	0	1	0		3	3	3	3
26	1	1		3	3	3	3		3	3	3	3
27	1	0		2	2	2	2		1	1	1	1
28	0	1		0	0	0	0		2	2	2	2
29	0	1		0	0	0	0		3	3	3	3
30	0	1		0	0	0	0		3	3	3	3
31	0	1		0	0	0	0		2	2	2	2
32	1	0		3	3	3	3		1	1	1	1
33	1	1		3	3	2	3		2	2	2	2
34	0	0		0	0	0	0		1	1	1	1
35	1	1		2	2	2	2		3	3	3	3
36	1	1		2	3	2	2		3	3	2	3
37	0	0		1	0	0	0		0	0	0	0
38	1	0		2	2	2	2		1	1	1	1
39	1	1		2	2	2	2		2	2	2	2
40	0	1		0	1	1	1		2	2	2	2
41	1	1		3	3	2	3		3	2	2	2
42	1	1		2	2	2	2		2	2	2	2
43	1	1		3	2	3	3		3	3	3	3
44	0	1		0	0	0	0		3	3	3	3
45	0	1		2	1	1	1		3	3	2	3
46	0	1		1	1	0	1		2	3	3	3
47	0	0		0	0	0	0		1	1	1	1
48	1	1		3	3	3	3		3	3	3	3
49	1	1		2	2	2	2		2	2	2	2
50	1	1		2	3	2	2		2	3	2	2
51	0	1		0	0	0	0		3	3	3	3
52	1	1		3	3	3	3		3	3	3	3
53	0	1		1	1	1	1		2	2	2	2
54	0	0		1	1	1	1		2	2	2	2
55	1	1		3	3	3	3		2	2	2	2
